# GAML: genome assembly by maximum likelihood

**DOI:** 10.1186/s13015-015-0052-6

**Published:** 2015-06-03

**Authors:** Vladimír Boža, Broňa Brejová, Tomáš Vinař

**Affiliations:** Faculty of Mathematics, Physics, and Informatics, Comenius University, Mlynská dolina, 842 48 Bratislava, Slovakia

**Keywords:** Genome assembly, Maximum likelihood, Simulated annealing, De Bruijn graphs, Next generation sequencing

## Abstract

**Background:**

Resolution of repeats and scaffolding of shorter contigs are critical parts of genome assembly. Modern assemblers usually perform such steps by heuristics, often tailored to a particular technology for producing paired or long reads.

**Results:**

We propose a new framework that allows systematic combination of diverse sequencing datasets into a single assembly. We achieve this by searching for an assembly with the maximum likelihood in a probabilistic model capturing error rate, insert lengths, and other characteristics of the sequencing technology used to produce each dataset. We have implemented a prototype genome assembler GAML that can use any combination of insert sizes with Illumina or 454 reads, as well as PacBio reads. Our experiments show that we can assemble short genomes with N50 sizes and error rates comparable to ALLPATHS-LG or Cerulean. While ALLPATHS-LG and Cerulean require each a specific combination of datasets, GAML works on any combination.

**Conclusions:**

We have introduced a new probabilistic approach to genome assembly and demonstrated that this approach can lead to superior results when used to combine diverse set of datasets from different sequencing technologies. Data and software is available at http://compbio.fmph.uniba.sk/gaml.

## Background

The second and third generation sequencing technologies have dramatically decreased the cost of sequencing. Nowadays, we have a surprising variety of sequencing technologies, each with its own strengths and weaknesses. For example, Illumina platforms are characteristic by low cost and high accuracy, but the reads are short. On the other hand, Pacific Biosciences offer long reads at the cost of quality and coverage. In the meantime, the cost of sequencing was brought down to the point, where it is no longer a sole domain of large sequencing centers; even small labs can experiment with cost-effective genome sequencing. As a result, it is not realistic to assume an existence of a single standard protocol for sequencing genomes of a particular size. In this paper, we propose a framework for genome assembly that allows flexible combination of datasets from different technologies in order to harness their individual strengths.

Modern genome assemblers are usually based either on the overlap–layout–consensus framework (e.g. Celera [1], SGA [2]), or on de Bruijn graphs (e.g. Velvet [3], ALLPATHS-LG [4]). Both approaches can be seen as special cases of a string graph [5], in which we represent sequence fragments as vertices, while edges represent possible adjacencies of fragments in the assembly. A genome assembly is simply a set of walks through this graph. The main difference between the two frameworks is how we arrive at a string graph: through detecting long overlaps of reads (overlap–layout–consensus) or through construction of de Bruijn graphs based on* k*-mers.

However, neither of these frameworks is designed to systematically handle pair-end reads and additional heuristic steps are necessary to build larger scaffolds from assembled contigs. For example, ALLPATHS-LG [4] uses libraries with different insert lengths for scaffolding contigs assembled without the use of paired read information, while Cerulean [6] uses Pacific Biosystems long reads for the same purpose. Recently, the techniques of paired de Bruijn graphs [7] and pathset graphs [8] were developed to address paired reads systematically, however these approaches cannot combine libraries with different insert sizes.

Combination of sequencing technologies with complementary strengths can help to improve assembly quality. However, it is not feasible to design new algorithms for every possible combination of datasets. Often it is possible to supplement previously developed tools with additional heuristics for new types of data. For example, PBJelly [9] uses Pacific Biosystems reads solely to aid gap filling in draft assemblies. Assemblers like PacbioToCa [10] or Cerulean [6] use short reads to improve the quality of Pacific Biosystems reads so that they can be used within traditional assemblers. However, such approaches do not use all information contained within the datasets.

We propose a new framework that allows systematic combination of diverse datasets into a single assembly, without requiring a particular type of data for specific heuristic steps. Recently, probabilistic models have been used very successfully to evaluate the quality of genome assemblers [11–13]. In our work, we use likelihood of a genome assembly as an optimization criterion, with the goal of finding the assembly with the highest likelihood. Even though this may not be always feasible, we demonstrate that optimization based on simulated annealing can be very successful at finding high likelihood genome assemblies.

To evaluate the likelihood, we adapted a model by Ghodsi et al. [13], which can capture characteristics of each dataset, such as sequencing error rate, as well as length distribution and expected orientation of paired reads (“Probabilistic model for sequence assembly”). We can thus transparently combine information from multiple diverse datasets into a single score. Previously, there have been several works in this direction in much simpler models without sequencing errors [14, 15]. These papers used likelihood to estimate repeat counts, without considering other problems, such as how exactly are repeats integrated within scaffolds.

To test our framework, we have implemented a prototype genome assembler genome assembly by maximum likelihood (GAML) that can use any combination of insert sizes with Illumina or 454 reads, as well as PacBio reads. The starting point of the assembly are short contigs derived from Velvet [3] with very conservative settings in order to avoid assembly errors. We then use simulated annealing to combine these short contigs into high likelihood assemblies (“Finding a high likelihood assembly”). We compare our assembler to existing tools on benchmark datasets (“Experimental evaluation”), demonstrating that we can assemble genomes of up to 10 MB long with N50 sizes and error rates comparable to ALLPATHS-LG or Cerulean. For larger genomes, we can start from an assembly given by a different tool and improve on the result. While ALLPATHS-LG and Cerulean each require a very specific combination of datasets, GAML works on any combination.

## Probabilistic model for sequence assembly

Recently, several probabilistic models were introduced as a measure of the assembly quality [11–13]. All of these authors have shown that the likelihood consistently favours higher quality assemblies. In general, the probabilistic model defines the probability $\Pr (R|A)$ that a set of sequencing reads* R* is observed assuming that assembly* A* is the correct assembly of the genome. Since the sequencing itself is a stochastic process, it is very natural to characterize concordance of reads and an assembly by giving a probability of observing a particular read. In our work, instead of evaluating the quality of a single assembly, we use the likelihood as an optimization criterion with the goal of finding high likelihood genome assemblies. We adapt the model of Ghodsi et al. [13], which we describe in this section.

### Basics of the likelihood model

The model assumes that individual reads are independently sampled, and thus the overall likelihood is the product of likelihoods of the reads: $\Pr (R|A) = \prod _{r\in R} \Pr (r|A).$ To make the resulting value independent of the number of reads in set* R*, we use as the main assembly score the log average probability of a read computed as follows: $\text {LAP}(A|R) = (1/|R|)\sum\nolimits_{r\in R} \log \Pr (r|A).$ Note that maximizing $\Pr (R|A)$ is equivalent to maximizing $\text {LAP}(A|R)$.

If the reads were error-free and each position in the genome was sequenced equally likely, the probability of observing read* r* would simply be $\Pr (r|A)=n_r/(2L)$, where $n_r$ is the number of occurrences of the read as a substring of the assembly *A*,* L* is the length of* A*, and thus 2*L* is the length of the two strands combined [14]. Ghodsi et al. [13] have shown a dynamic programming computation of read probability for more complex models, accounting for sequencing errors. The algorithm marginalizes over all possible alignments of* r* and* A*, weighting each by the probability that a certain number of substitution and indel errors would happen during sequencing. In particular, the probability of a single alignment with* m* matching positions and* s* errors (substitutions and indels) is defined as $R(s,m)/(2L)$, where $R(s,m) = \epsilon ^{s}(1-\epsilon )^{m}$ and $\epsilon$ is the sequencing error rate.

However, the full dynamic programming is too time consuming, and in practice only several best alignments contribute significantly to the overall probability. We approximate the probability of observing read* r* with an estimate based on a set* S*_*r*_ of a few best alignments of* r* to genome *A*, as obtained by one of the standard fast read alignment tools:

1 $$\begin{aligned} \Pr (r|A)\approx \frac{\sum _{j\in S_r} R(s_j, m_j)}{2L}, \end{aligned}$$

where* m*_*j*_ is the number of matches in the* j*th alignment, and* s*_*j*_ is the number of mismatches and indels implied by this alignment. The formula assumes the simplest possible error model, where insertions, deletions, and substitutions have the same probability, and ignores GC content bias. Of course, much more comprehensive read models are possible (see e.g. [12]).

### Paired reads

Many technologies provide paired reads produced from the opposite ends of a sequence insert of a certain size. We assume that the insert size distribution in a set of reads* R* can be modeled by the normal distribution with known mean* μ* and standard deviation* σ*. The probability of observing paired reads* r*_1_ and * r*_2_ can be estimated from the sets of alignments $S_{r_1}$ and $S_{r_2}$ as follows:

2 $$\begin{aligned} \Pr (r_1, r_2|A) \approx \frac{1}{2L} \displaystyle \sum _{j_1 \in S_{r_1}} \displaystyle \sum _{j_2 \in S_{r_2}} R(s_{j_1}, m_{j_1}) R(s_{j_2}, m_{j_2}) \Pr (d(j_1, j_2)|\mu , \sigma ) \end{aligned}$$

As before, $m_{j_i}$ and $s_{j_i}$ are the numbers of matches and sequencing errors in alignment* j*_*i*_ respectively, and $d(j_1,j_2)$ is the distance between the two alignments as observed in the assembly. If alignments * j*_1_ and * j*_2_ are in two different contigs, or on inconsistent strands, $\Pr (d(j_1, j_2)|\mu , \sigma )$ is zero.

### Reads that have no good alignment to A

Some reads or read pairs do not align well to* A*, and as a result, their probability $\Pr (r|A)$ is very low; our approximation by a set of high-scoring alignments can even yield zero probability if set* S*_*r*_ is empty. Such extremely low probabilities then dominate the log likelihood score. Ghodsi et al. [13] propose a method that assigns such a read a score approximating the situation when the read would be added as a new contig to the assembly. We modify their formulas for variable read length, and use score $e^{c + k\ell }$ for a single read of length $\ell$ or $e^{c + k(\ell _1 + \ell _2)}$ for a pair of reads of lengths $\ell _1$ and $\ell _2$. Values* k* and* c* are scaling constants set similarly as by Ghodsi et al. [13]. These alternative scores are used instead of the read probability $\Pr (r|A)$ whenever the probability is lower than the score.

### Multiple read sets

Our work is specifically targeted at a scenario, where we have multiple read sets obtained from different libraries with different insert lengths or even with different sequencing technologies. We use different model parameters for each set and compute the final score as a weighted combination of log average probabilities for individual read sets $R_1,\dots, R_k$:

3 $$\begin{aligned} \text {LAP}(A|R_1, \dots , R_k) = w_1 \text {LAP}(A|R_1) + \dots + w_k \text {LAP}(A|R_k). \end{aligned}$$

In our experiments, we use weight $w_i=1$ for most datasets, but we lower the weight for Pacific Biosciences reads, because otherwise they dominate the likelihood value due to their longer length. The user can also increase or decrease weights * w*_*i*_ of individual sets based on their reliability.

### Penalizing spuriously joined contigs

The model described above does not penalize obvious misassemblies when two contigs are joined together without any evidence in the reads. We have observed that to make the likelihood function applicable as an optimization criterion for the best assembly, we need to introduce a penalty for such spurious connections. We say that a particular base* j* in the assembly is *connected* with respect to read set* R* if there is a read which covers base* j* and starts at least* k* bases before* j*, where* k* is a constant specific to the read set. In this setting, we treat a pair of reads as one long read. If the assembly contains* d* disconnected bases with respect to* d*, penalty $\alpha d$ is added to the $\text {LAP}(A|R)$ score (*α* is a scaling constant).

### Properties of different sequencing technologies

Our model can be applied to different sequencing technologies by appropriate settings of model parameters. For example, Illumina technology typically produces reads of length 75–150 bp with error rate below 1% [16]. For smaller genomes, we often have a high coverage of Illumina reads. Using paired reads or mate pair technologies, it is possible to prepare libraries with different insert sizes ranging up to tens of kilobases, which are instrumental in resolving longer repeats [4]. To align these reads to proposed assemblies, we use Bowtie2 [17]. Similarly, we can process reads by the Roche 454 technology, which are characteristic by higher read lengths (hundreds of bases).

Pacific Biosciences technology produces single reads of variable length, with median length reaching several kilobases, but the error rate exceeds 10% [6, 16]. Their length makes them ideal for resolving ambiguities in assemblies, but the high error rate makes their use challenging. To align these reads, we use BLASR [18]. When we calculate the probability $\Pr (r|A)$, we consider not only the best alignments found by BLASR, but for each BLASR alignment, we also add probabilities of similar alignments in its neighborhood. More specifically, we run a banded version of the forward algorithm by [13], considering all alignments in a band of size three around a guide alignment produced by BLASR.

## Finding a high likelihood assembly

Complex probabilistic models, like the one described in “Probabilistic model for sequence assembly”, were previously used to compare the quality of several assemblies [11–13]. In our work, we instead attempt to find the highest likelihood assembly directly. Of course, the search space is huge, and the objective function too complex to admit exact methods. Here, we describe an effective optimization routine based on the simulated annealing framework [19].

Our algorithm for finding the maximum likelihood assembly consists of three main steps: preprocessing, optimization, and postprocessing. In *preprocessing*, we decrease the scale of the problem by creating an assembly graph, where vertices correspond to contigs and edges correspond to possible adjacencies between contigs supported by reads. In order to make the search viable, we will restrict our search to assemblies that can be represented as a set of walks in this graph. Therefore, the assembly graph should be built in a conservative way, where the goal is not to produce long contigs, but rather to avoid errors inside them. In the *optimization step*, we start with an initial assembly (a set of walks in the assembly graph), and iteratively propose changes in order to optimize the assembly likelihood. Finally, *postprocessing* examines the resulting walks and splits some of them into shorter contigs if there are multiple equally likely possibilities of resolving ambiguities. This happens, for example, when the genome contains long repeats that cannot be resolved by any of the datasets. In the rest of this section, we discuss individual steps in more detail.

### Optimization by simulated annealing

To find a high likelihood assembly, we use an iterative simulated annealing scheme. We start from an initial assembly $A_0$ in the assembly graph. In each iteration, we randomly choose a *move* that proposes a new assembly $A'$ similar to the current assembly* A*. The next step depends on the likelihoods of the two assemblies* A* and $A'$ as follows:

- If $\text {LAP}(A'|R)\ge \text {LAP}(A|R)$, the new assembly $A'$ is accepted and the algorithm continues with the new assembly.
- If $\text {LAP}(A'|R)< \text {LAP}(A|R)$, the new assembly $A'$ is accepted with probability $e^{(\text {LAP}(A'|R)-\text {LAP}(A|R))/T}$; otherwise $A'$ is rejected and the algorithm retains the old assembly* A* for the next step.

Here, parameter* T* is called the temperature, and it changes over time. In general, the higher the temperature, the more aggressive moves are permitted. We use a simple cooling schedule, where $T = T_0/\ln (i)$ in the* i*th iteration. The computation ends when there is no improvement in the likelihood for a certain number of iterations. We select the assembly with the highest LAP score as the result.

To further reduce the complexity of the assembly problem, we classify all contigs as either *long* (more than 500 bp) or *short* and concentrate on ordering the long contigs correctly. The short contigs are used to fill the gaps between the long contigs. Recall that each assembly is a set of walks in the assembly graph. A contig can appear in more than one walk or can be present in a single walk multiple times.

**Figure 1:**
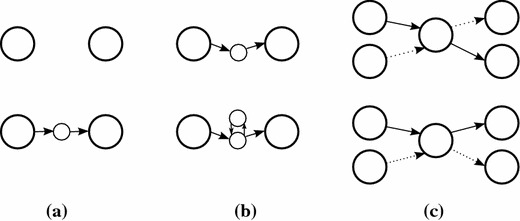
Examples of proposal moves.** a** Walk extension joining two walks.** b** Local improvement by addition of a new loop.** c** Repeat interchange.

Proposals of new assemblies are created from the current assembly using the following moves:

- *Walk extension* (Figure 1a) We start from one end of an existing walk and randomly walk through the graph, in every step uniformly choosing one of the edges outgoing from the current node. Each time we encounter the end of another walk, the two walks are considered for joining. We randomly (uniformly) decide whether we join the walks, end the current walk without joining, or continue walking.
- *Local improvement* (Figure 1b) We optimize the part of some walk connecting two long contigs* s* and* t*. We first sample multiple random walks starting from contig* s*. In each walk, we only consider nodes from which contig* t* is reachable. Then we evaluate these random walks and choose the one that increases the likelihood the most. If the gap between contigs * s* and * t* is too big, we instead use a greedy strategy where in each step we explore multiple random extensions of the walk of length around 200 bp and pick the one with the highest score.
- *Repeat optimization* We optimize the copy number of short tandem repeats. We do this by removing or adding a loop to some walk. We precompute the list of all short loops (up to five nodes) in the graph and use it for adding loops.
- *Joining with advice* We join two walks that are spanned by long reads or paired reads with long inserts. We first select a starting walk, align all reads to this walk and randomly choose a read which has the other end outside the walk. Then we find to which node this other end belongs to and join appropriate walks. If possible, we fill the gap between the two walks using the same procedure as in the local improvement move. Otherwise we introduce a gap filled with Ns.
- *Disconnecting* We remove a path through short contigs connecting two long contigs in the same walk, resulting in two shorter walks.
- *Repeat interchange* (Figure 1c) If a long contig has several incoming and outgoing walks, we optimize the pairing of incoming and outgoing edges. In particular, we evaluate all moves that exchange parts of two walks through this contig. If one of these changes improves the score, we accept it and repeat this step, until the score cannot be improved at this contig.

At the beginning of each annealing step, the type of the move is chosen randomly; each type of move has its own probability. We also choose randomly the contig at which we attempt to apply the move.

Note that some moves (e.g. local improvement) are very general, while other moves (e.g. joining with advice) are targeted at specific types of data. This does not contradict a general nature of our framework; it is possible to add new moves as new types of data emerge, leading to improvement when using specific datasets, while not affecting the performance when such data is unavailable.

### Preprocessing and the initial assembly

To obtain the assembly graph, we use Velvet with basic error correction and unambiguous concatenation of* k*-mers. These settings will produce very short contigs, but will also give a much lower error rate than a regular Velvet run. GAML with the default settings then uses each long contig as a separate walk in the starting assembly for the simulated annealing procedure.

### Postprocessing

The assembly obtained by the simulated annealing procedure may contain walks with no evidence for a particular configuration of incoming and outgoing edges in the assembly graph. This happens for example if a repeat is longer than the span of the longest paired read. In this case, there would be several versions of the assembly with the same or very similar likelihood score. In the postprocessing step, we therefore apply the repeat interchange move at every possible location of the assembly. If the likelihood change resulting from such a move is negligible, we break the corresponding walks into shorter contigs to avoid assembly errors.

### Fast likelihood evaluation

The most time consuming step in our algorithm is evaluation of the assembly likelihood, which we perform in each iteration of simulated annealing. This step involves alignment of a large number of reads to the current assembly. However, only a small part of the assembly is changed in each annealing step, which we can use to significantly reduce the running time. Next, we describe three optimizations implemented in our software.

*Limiting read alignment to affected regions of the assembly* Since only a small portion of the assembly is affected in each step, we can keep most alignments from the previous iterations and only align reads to the regions that changed. To determine these regions, we split walks into overlapping windows, each window containing several adjacent contigs of a walk. Windows should be as short as possible, but adjacent windows should overlap by at least $2\ell _r$ bases, where $\ell _r$ is the length of the longest read. As a result, each alignment is completely contained in at least one window even in the presence of extensive indels.

We determine the window boundaries by a simple greedy strategy, which starts at the first contig of a walk, and then extends the window by at least $2\ell _r$ bases beyond the boundary of the first contig. The next window always starts at the latest possible location that ensures a sufficient overlap and extends at least $2\ell _r$ bases beyond the end of the previous window.

For each window, we keep the position and edit distance of all alignments. In each annealing step, we identify which windows of the assembly were changed since the last iteration. We then glue together overlapping windows and align reads against these sequences.

We further improve this heuristics by avoiding repeated alignments of reads to interiors of long contigs, because these parts of the assembly never change. In particular, if some window starts with a long contig, we only realign reads to the last $2\ell _r$ bases from that contig, and similarly we use only the first $2\ell _r$ bases from a long contig at the end of a window.

*Reducing the number of reads which need to be aligned* The first improvement eliminates most of the assembly from read mapping. In contrast, the second improvement reduces the set of reads which need to be realigned, because most of the reads will not align to the changed part of the assembly. We use a prefiltering step to find the reads which are likely to align to the target sequence. In the current implementation, we use the following three options for such filtering.

In the simplest approach, we look for reads which contain some* k*-mer (usually $k=13$) from the target sequence. We store an index of all* k*-mers from all reads in a hash map. In each annealing step, we iterate over all* k*-mers in the target portion of the assembly and retrieve reads that contain them. This approach is very memory consuming, because the identifier of each read is stored for each* k*-mer from this read.

In the second approach, we save memory using min-hashing [20]. Given hash function* h*, the min-hash of set* A* is defined as $m(A) = \min _{x \in A} h(x)$. For each read* R*, we calculate min-hash for the set of all its* k*-mers. Thus, the identifier of each read is stored in the hash table only once. In each annealing step, we calculate the min-hash for each substring of the target sequence of length $\ell _r$ and retrieve the reads that have the same min-hash.

An important property of min-hashing is that $\Pr (m(A) = m(B)) = J(A, B)$, where $J(A, B) = \frac{|A \cap B|}{|A \cup B|}$ is the Jaccard similarity of two sets* A* and* B* [21]. The statement holds if the hash function* h* is randomly chosen from a family with the min-wise independence property, which means that for every subset of elements* X*, each element in* X* has the same chance to have the minimum hash.

Note that strings with a very small edit distance have a high Jaccard similarity between their* k*-mer sets, and therefore a high chance that they will hash to the same value using min-hashing. We can use several min-hashes with different hash functions to improve the sensitivity of our filtering at the cost of additional memory.

In our implementation, we use a simple hash function which maps* k*-mers into 32-bit integers. We first represent the* k*-mer as an integer (where each base corresponds to two bits). We then xor this integer with a random number. Finally, we perform mixing similar to the finalization of the Murmur hash function [22]: 

We choose this finalizer because the Murmur hash function is fast and results in few collisions. It is not min-hash independent, but we found it to perform well in practice.

To illustrate the specificity and sensitivity of min-hashing, we have compared our min-hashing approach with indexing all* k*-mers (with $k=15$) on evaluating LAP of the Allpaths-LG assembly of *Staphylococus aureus* (using read set SA1 described in “Experimental evaluation” and aligning it to the whole *S. aureus* genome). Indexing all* k*-mers resulted in 3,659,273 alignments found by examining 21,241,474 candidate positions. Using min-hashing with three hash functions, we were able to find 3,639,625 alignments by examining 3,905,595 candidates positions. Since these reads have a low error rate,* k*-mer indexing retrieves practically all relevant alignments, while the sensitivity of min-hashing is approximately 99.5%. In min-hashing, 93% of examined positions yield an alignment, whereas specificity of* k*-mer indexing is only 17%. Also min-hashing used 30 times smaller index.

Note that min-hashing was previously used in a similar context by Berlin et al. [23] to find similarities among PacBio reads. However, since PacBio reads have a high error rate, the authors had to use a high number of hash functions, whereas we use only a few hash functions to filter Illumina reads, which have a low error rate.

In GAML, we filter PacBio reads by a completely different approach, which is based on alignments, rather than* k*-mers. In particular, we take all reasonably long contigs (at least 100 bases) and align them to PacBio reads. Since BLASR can find alignments where a contig and a read overlap by only around 100 bases, we use these alignments as a filter.

*Final computation of the likelihood score* When all reads are properly aligned to the new version of the assembly, we can combine the alignments to the final score. In the implementation, we need to handle several issues, such as correctly computing likelihood for reads that align to multiple walks, assigning a special likelihood to reads without any good alignment, and avoiding double counting for reads that align to regions covered by two overlapping windows of the same walk.

Again we improve the running time by considering only reads that were influenced by the most recent change. Between consecutive iterations, we keep all alignments for each sequence window of the assembly and recompute only alignments to affected windows, as outlined above. We also keep the likelihood value of each read or a read pair. Recall that the likelihood of a read or a read pair is the sum of likelihoods of individual alignments.

In each iteration, we then identify which walks were removed and added. Then we calculate likelihoods of all read alignments in these walks (using stored or newly computed alignments) and we use these values to adjust the likelihood values of individual reads, subtracting for removed walks and adding for new walks. At this step, we also handle paired reads, identifying pairs of alignments in correct distance and orientation. Finally, we sum likelihoods of all reads in each dataset and compute the total likelihood score.

## Experimental evaluation

We have implemented the algorithm proposed in the previous section in a prototype assembler GAML. At this stage, GAML can assemble small genomes (approx. 10 Mbp) in a reasonable amount of time (approximately 4 h on a single CPU and using 10GB of memory).

To evaluate the quality of our assembler, we have adopted the methodology used for Genome Assembly Gold-Standard Evaluation [24], using metrics on scaffolds. We have used the same genomes and libraries as Salzber et al. [24] (the *Staphylococus aureus* genome and the human chromosome 14) and Deshpande et al. [6] (the *Escherichia coli* genome). The overview of the datasets is shown in Table 1. An additional dataset EC3 (long insert, low coverage) was simulated using the ART software [25].

We have evaluated GAML in the following scenarios:

1. combination of fragment and short insert Illumina libraries (SA1, SA2),
2. combination of a fragment Illumina library and a long-read high-error-rate Pacific Biosciences library (EC1, EC2),
3. combination of a fragment Illumina library, a long-read high-error-rate Pacific Biosciences library, and a long jump Illumina library (EC1, EC2, EC3),

**Table 1:** Properties of datasets used

ID	References	Technology	Insert length (bp)	Read length (bp)	Coverage	Error rate (%)
*Staphylococus aureus* (2.87 Mbp)						
SA1	[24]	Illumina	180	101	90	3
SA2	[24]	Illumina	3,500	37	90	3
*Escherichia coli* (4.64 Mbp)						
EC1	[6]	Illumina	300	151	400	0.75
EC2	[6]	PacBio		4,000	30	13
EC3	Simulated	Illumina	37,000	75	0.5	4
*Human chromosome 14* (88.29 Mbp)						
H1	[24]	Illumina	150	101	42	1
H2	[24]	Illumina	2,500	101	26	3
H3	[24]	Illumina	35,000	76	1.3	4.5

In each scenario, we use the short insert Illumina reads (SA1 or EC1) in Velvet with conservative settings to build the initial contigs and assembly graph. For the LAP score, we give all Illumina datasets weight 1 and the PacBio dataset weight 0.01. The results are summarized in Table 2. Note that none of the assemblers considered here can effectively run in all three of these scenarios, except for GAML.

**Table 2:** Comparison of assembly accuracy in the first three scenarios

Assembler	Number of scaffolds	Longest scaffold (kb)	Longest scaffold corr. (kb)	N50 (kb)	Err.	N50 corr. (kb)	LAP
*Staphylococus aureus*, read sets SA1, SA2							
GAML	28	1,191	1,191	514	*0*	514	*−23.45*
Allpaths-LG	*12*	1,435	*1,435*	1,092	*0*	*1,092*	−25.02
SOAPdenovo	99	518	518	332	*0*	332	−25.03
Velvet	45	958	532	762	17	126	−25.34
Bambus2	17	1,426	1,426	1,084	*0*	1,084	−25.73
MSR-CA	17	*2,411*	1,343	*2,414*	3	1,022	−26.26
ABySS	246	125	125	34	1	28	−29.43
Cons. Velvet*	219	95	95	31	*0*	31	−30.82
SGA	456	286	286	208	1	208	−31.80
*Escherichia coli*, read sets EC1, EC2							
PacbioToCA	55	1,533	1,533	*957*	*0*	*957*	*−33.86*
GAML	29	1,283	1,283	653	*0*	653	−33.91
Cerulean	*21*	*1,991*	*1,991*	694	*0*	694	−34.18
AHA	54	477	477	213	5	194	−34.52
Cons. Velvet*	383	80	80	21	*0*	21	−36.02
*Escherichia coli*, read sets EC1, EC2, EC3							
GAML	*4*	*4,662*	*4,661*	*4,662*	*3*	*4,661*	*−60.38*
Celera	19	4,635	2,085	4,635	19	2,085	−61.47
Cons. Velvet*	383	80	80	21	*0*	21	−72.03

For all assemblies, N50 values are based on the actual genome size. All misjoins were considered as errors and error-corrected values of N50 and contig sizes were obtained by breaking each contig at each error [24]. All assemblies except for GAML and conservative Velvet were obtained from [24] in the first experiment, and from [6] in the second experiment.

Italic numbers in each column signify the best result.

* Velvet with conservative settings used to create the assembly graph in our method.

In the first scenario, GAML performance ranks third among zero-error assemblers in the N50 length. The best N50 assembly is given by ALLPATHS-LG [4]. A closer inspection of the assemblies indicates that GAML missed several possible joins. One such miss was caused by a 4.5 kbp repeat, while the longest insert size in this dataset is 3.5 kbp. Even though in such cases it is sometimes possible to reconstruct the correct assembly thanks to small differences in the repeated regions, the difference in likelihood between alternative repeat resolutions may be very small. Another missed join was caused by a sequence coverage gap penalized in our scoring function. Perhaps in both of these cases the manually set constants may have caused GAML to be overly conservative. Otherwise, the GAML assembly is very similar to the one given by ALLPATHS-LG.

In the second scenario, Pacific Biosystems reads were employed instead of jump libraries. These reads pose a significant challenge due to their high error rate, but they are very useful due to their long length. Assemblers such as Cerulean [6] deploy special algorithms taylored to this technology. GAML, even though not explicitly tuned to handle Pacific Biosystems reads, builds an assembly with N50 size and the number of scaffolds very similar to that of Cerulean. In N50, both programs are outperformed by PacbioToCA [10], however, this is again due to a few very long repeats (approx. 5,000 bp) in the reference genome which were not resolved by GAML or Cerulean. (Cerulean also aims to be conservative in repeat resolution.) Note that in this case, simulated annealing failed to give the highest likelihood assembly among those that we examined, so perhaps our results can be improved by tuning the likelihood optimization.

The third scenario shows that the assembly quality can be hugely improved by including a long jump library, even if the coverage is really small (we have used 0.5× coverage in this experiment). This requires a flexible genome assembler; in fact, only Celera [1] can process this data, but GAML assembly is clearly superior. We have attempted to run also ALLPATHS-LG, but the program could not process this combination of libraries. Compared to the previous scenario, GAML N50 size increased approximately sevenfold (or approx. fourfold compared to the best N50 from the second scenario assemblies).

**Table 3:** Improving existing assemblies of the human chromosome 14 by GAML

Assembler	Number of scaffolds	Longest scaffold (kb)	Longest scaffold corr. (kb)	N50 (kb)	Err.	N50 corr. (kb)	LAP
*Human chromosome 14*, starting from Velvet assembly							
Before	1,081	4,628	263	1,190	9,156	27	−138.765779
After	1,634	1,046	265	347	8,049	27	−138.632657
REAPR	17,727	153	81	36	4,607	14	−162.869192
*Human chromosome 14*, starting from ALLPATHS assembly							
Before	129	81,640	14,918	81,640	34	7,652	−111.288806
After	139	81,640	14,918	81,640	33	7,652	−111.287938
REAPR	858	977	146	190	4,230	17	−168.024865

In both experiments, we use read sets H1, H2, and H3 and compare the original assembly computed by another tool with the assembly found by GAML.

## Improving previously assembled genomes

For medium and large genomes, it would take GAML too many iterations to arrive at a reasonable assembly starting from the contigs produced by Velvet with conservative settings. However, it is still possible to scale up GAML to larger genomes by using another assembler to provide a more reasonable starting point.

To this end, we have to map such an input assembly to the assembly graph. We first align the assembly contigs to the Velvet contigs using NUCmer [26]. We keep only alignments which cover entire Velvet contigs and have a high sequence identity. If a single input contig is aligned to several Velvet contigs, we connect these Velvet contigs to a walk in the assembly graph. The missing portions of the walk are found by dynamic programming so as to minimize the edit distance between the input contig and the walk. In the dynamic programming, we consider only edit distance of up to 10, and if we do not find a connection within this threshold, we add a corresponding number of Ns to our walk.

If the input assembly differs too much from the Velvet contigs, a good mapping of the contigs to walks in the Velvet assembly graph cannot be found. In such cases, we construct the assembly graph directly from the input assembly. We first build a deBruijn graph from the contigs, and then we concatenate nodes connected by unambiguous connections.

We can now use GAML to improve medium-size genome assemblies (approx. 100 Mbp). In this setting, 10,000 iterations require approximately 2 days time and 50GB of memory.

We have tested this approach by using Illumina reads with three different insert sizes (H1, H2, H3) on the human chromosome 14 (data from [24]; see Table 1). We use the non-conservative Velvet assembly and the ALLPATHS assembly as our starting point. The results are shown in Table 3.

Starting from the Velvet assembly, GAML makes 787 breaks and 234 joins, reducing the error count by more than a thousand. Our joins did not introduce any new errors to the assembly. The ALLPATHS assembly has a much higher quality, and starting from this assembly, GAML decreases the number of errors only by one at the cost of introducing ten breaks. In both cases, we were able to remove some assembly errors, while not decreasing the error-corrected N50 values. Perhaps more corrections could be found if we ran our algorithm for more iterations (especially in the Velvet case).

Since breaks predominate in the changes made by GAML, we have also compared our results to REAPR [27], which is a tool that aligns reads to an existing assembly and then splits contigs at the positions weakly supported or even in conflict with the reads. When it concludes that some place is not a breakpoint, but should instead contain an insertion, it inserts a sequence of Ns. Note that REAPR can only process one jumping library along with an optional fragment library, and it requires the library to have a reasonable coverage (15×). Due to these constraints, we have used REAPR only with short jump library H2. For the Velvet assembly, REAPR removes significantly more errors than GAML, but at the cost of a great increase in the number of contigs and a decrease in the error-corrected N50 value. REAPR also introduces many cuts in the ALLPATHS assembly and the GAGE error checking tools report a high increase in errors. We hypothesize that this due to REAPR adding many regions of Ns in the corrected assembly, which leads to a high number of small contigs which GAGE checker cannot align correctly.

## Conclusion

We have presented a new probabilistic approach to genome assembly, maximizing likelihood in a model capturing essential characteristics of individual sequencing technologies. It can be used on any combination of read datasets and can be easily adapted to other technologies arising in the future. We have also adapted our tool to improve existing assemblies after converting a given assembly to a set of walks.

Our work opens several avenues for future research. First, we plan to further improve running time and memory and to allow the use of our tool on larger genomes. Second, the simulated annealing procedure could be improved by optimizing probabilities of individual moves or devising new types of moves. Finally, it would be interesting to explore even more detailed probabilistic models, featuring coverage biases and various sources of experimental error.
